# *Procambarus virginalis* Lyko, 2017: A new threat to Iberian inland waters

**DOI:** 10.1002/ece3.11362

**Published:** 2024-05-20

**Authors:** Omar Sánchez, Francisco J. Oficialdegui, Antonio Torralba‐Burrial, Ramón Arbesú, José María Valle‐Artaza, Ángel Fernández‐González, Alba Ardura, Andrés Arias

**Affiliations:** ^1^ Department of Organisms and Systems Biology (Zoology) University of Oviedo Oviedo Spain; ^2^ Faculty of Fisheries and Protection of Waters, South Bohemian Research Center of Aquaculture and Biodiversity of Hydrocenoses University of South Bohemia in České Budějovice Vodňany Czech Republic; ^3^ Institute of Natural Resources and Territorial Planning (Indurot) – University of Oviedo Mieres Spain; ^4^ Sección de Recursos Naturales, Servicio de Vida Silvestre Dirección General de Planificación Agraria, Consejería de Medio Rural y Política Agraria, Gobierno del Principado de Asturias Oviedo Spain; ^5^ Biosfera Consultoría Medioambiental S.L. Oviedo Spain; ^6^ Department of Functional Biology (Genetic) University of Oviedo Oviedo Spain; ^7^ University Centre for Water Research and Development (CUIDA) – University of Oviedo Mieres Spain

**Keywords:** crayfish, invasive species, marbled crayfish, *Procambarus fallax*, Spain

## Abstract

An eco‐monitoring programme to assess faunal biodiversity in the main rivers of the northern Iberian Peninsula (Spain) reveals the first occurrence of the marbled crayfish *Procambarus virginalis* (Decapoda: Cambaridae) in Iberian inland waters. Iberian specimens have been identified by combining morphological and genetic traits. We discuss the most plausible pathways and introduction vectors, its potential invasiveness and subsequent impacts on host localities. Our preliminary results raise concern about the potential threat of *P. virginalis* to native fauna and ecosystem dynamics, as *P. virginalis* was found in an area of great cultural and ecological importance with relevant populations of endangered species. Due to the invasive history of the marbled crayfish, eradication of these individuals is urgent. This study confirms the importance of early warning systems for exotic species, keeping the population, forest guards and field technicians informed about potential invasive species to execute a rapid and effective response.

## Graphical abstract

We provide the first record of *Procambarus virginalis* in the Iberian Peninsula, based on morphological and genetic evidence (molecular barcoding of COI). We discuss its most plausible introduction pathways, update its global geographical distribution, highlight the potential impacts if it becomes invasive in Iberia and recommend some proposal management practices.

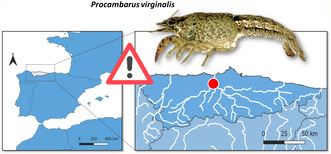

## 1 INTRODUCTION

Crayfish are the largest freshwater invertebrate organisms that form a very diverse group consisting of around 700 species (Crandall & De Grave, 2017). They can inhabit virtually all types of freshwater bodies and play important ecological roles – considered as ecosystem engineer species (Arias & Torralba‐Burrial, 2021; Reynolds et al., 2013). Over the last few decades, non‐native freshwater crayfish have become a prominent group in the study of biological invasions on a global scale. Their importance as a fishing and commercial resource, ornamental species in pet trade and their unique biological and ecological characteristics have led to their spread around the world due to human action (Lodge et al., 2012). The introduction of these ecosystem engineer species outside of their native range often causes serious ecological (Emery‐Butcher et al., 2020) and economic losses (Kouba et al., 2022). They can unleash direct negative impacts on native species or indirect ones mediated by pathogens, altering the functioning of river ecosystems (Chiatante et al., 2021).

In the Iberian Peninsula, five intentionally introduced crayfish species have formed non‐native established populations in the wild: *Procambarus clarkii*, *Pacifastacus leniusculus*, *Cherax quadricarinatus*, *Cherax destructor* and *Faxonius limosus* (Acevedo‐Limón et al., 2020; Arias & Torralba‐Burrial, 2021; Benejam et al., 2011; Vedia & Miranda‐Ferreiro, 2013). Furthermore, other crayfish species have been introduced in the past but failed to establish populations in the wild (e.g. *Astacus astacus*, *Pontastacus leptodactylus* or *Procambarus zonangulu*s). In addition, the European species *Austropotamobius fulcisianus* (previously referred to as *A. italicus* or *A. pallipes*) is considered a cryptogenic species (sensu Carlton, 1996) in Spain, due to the still lack of consensus among the scientific community on its status (Clavero et al., 2016; Clavero & Centeno‐Cuadros, 2016; Martínez‐Ríos et al., 2023; Matallanas et al., 2016).

The marbled crayfish or marmorkrebs, *Procambarus virginalis* Lyko, 2017, is a species described based on individuals kept in a German aquarium (Scholtz et al., 2003) without a clear taxonomic identification for several years (Marzano et al., 2009). *Procambarus virginalis* is unique for being the only obligate parthenogenetic decapod known to date, and therefore, no male individuals exist, and populations consequently consist of females (Scholtz et al., 2003; Vogt, 2008). This species had been previously identified as belonging to a new form of the North American species *Procambarus fallax* (Hagen, 1870), named *Procambarus fallax* f. *virginalis* (Martin et al., 2010). However, subsequent studies revealed substantial different fitness traits, reproductive incompatibility and genetic differences with *P. fallax* to be considered as an independent new species (Lyko, 2017).

Here, we report on the occurrence of the marbled crayfish, *P. virginalis*, in northern Spain, constituting the first record of this exotic species in the Iberian Peninsula. We describe the Spanish specimens based on morphological and genetic evidence (molecular barcoding of COI), discuss its most plausible introduction pathways and update its geographical distribution in Europe. Finally, we highlight the potential impacts it may cause if becomes invasive and recommend some proposal management practices.

## 2 MATERIALS AND METHODS

### 2.1 Field sampling and procedures

**FIGURE 1:**
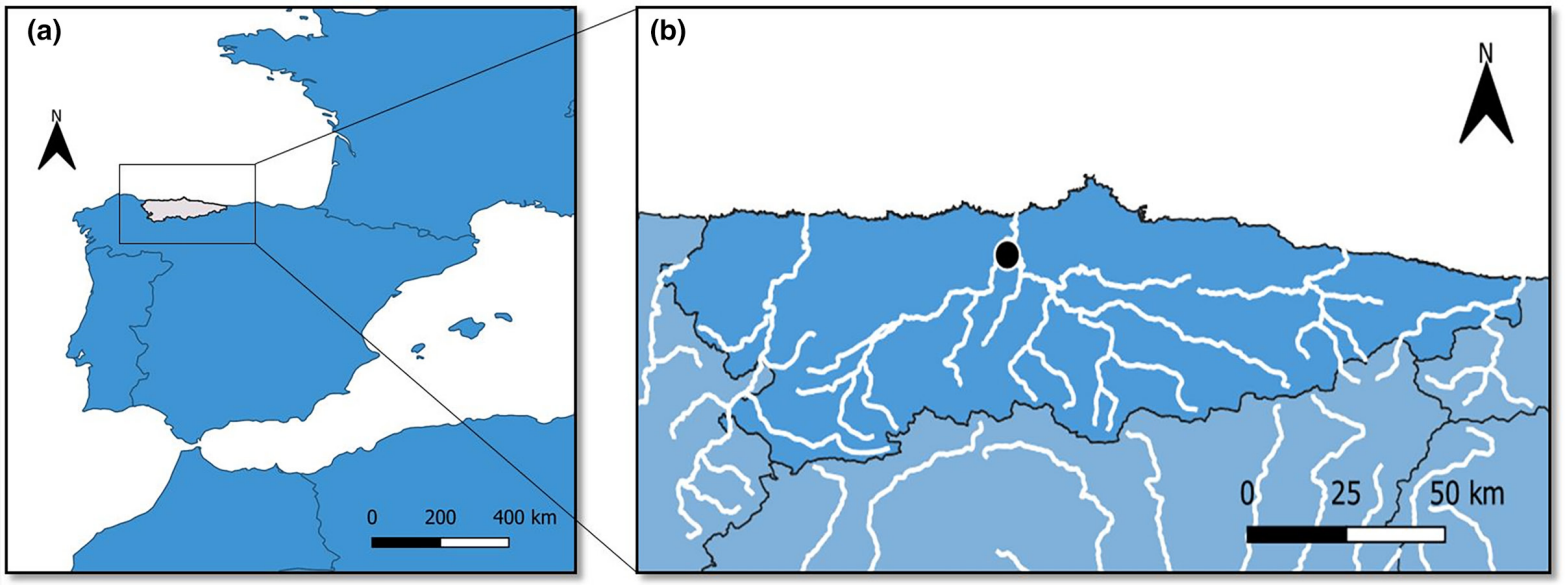
Geographical situation of the Principality of Asturias (grey) in the Iberian Peninsula (a) and the location where individuals of *Procambarus virginalis* were found, with the main river network highlighted (b).

During an assessment sampling of a river restoration project by the Cantabrian Hydrographic Confederation in the Nalón River, a total of four female specimens of presumed *P. virginalis* were collected, among several *P. clarkii* ones, from monthly catches from October to December 2022 near Santoseso, Nalón River (43°27′30″ N, 06°05′56″ W, 15 m, Candamo, Asturias, Spain) (Figure 1). Specimens were collected through electro‐fishing by personnel of an environmental consultant (Biosfera S.L.) and the Government of Asturias. Specimens were brought to the laboratory and photographed by a Canon EOS 1200D Digital SLR Camera with EF‐S 18–55 mm f/3.5–5.6 III lens. Their total length (carapace length (from the tip of the rostrum to the end of carapace) + abdomen length) was measured and other taxonomical features were examined under a Leica MZ125 high‐performance dissecting stereomicroscope. Subsequently, they were refrigerated in 70% ethanol and two of them were deposited at the Collection of the Department of Organisms and Systems (BOS) of the University of Oviedo (https://bos.uniovi.es/).

Maps were generated with QGIS 3.16.9 software. Information about *P. virginalis* geographical records was obtained from the literature and the Marmorkrebs.org https://sites.google.com/view/marmorkrebs/.

### 2.2 DNA extraction, PCR amplification and sequencing

DNA was extracted from 20 to 50 mg of ethanol‐preserved muscle tissue obtained from the leg appendices of one of the four studied specimens, using E.Z.N.A. Tissue DNA Kit (Omega Bio‐Tek) and following the manufacturer's protocol. A fragment of the cytochrome *c* oxidase subunit I (COI) mitochondrial gene, which displays great variability among species and very low within species (Filonzi et al., 2023), was amplified using the universal primers LCO1490 and HCO2198 (Folmer et al., 1994) by means of polymerase chain reaction (PCR) in a total reaction volume of 40 μL. The reaction mixture contained 2.5 μL template DNA, 2.5 μL of 25 mM MgCl_2_, 4 μL of 2.5 mM dNTPs, 1 μL of 10 μM primers, 0.15 μL Taq polymerase (GoTaq® G2 Flexi DNA Polymerase 5 U/μL) and 8 μL of 5x Buffer GoTaq® Promega (1× final concentration). PCR conditions consisted of an initial denaturation step of 95°C for 4 min, followed by 35 amplification cycles (95°C for 45 s, 48°C for 45 s and 72°C for 30 s) and a final elongation step at 72°C for 7 min. The resulting PCR product was checked for good amplification in horizontal electrophoresis (2% agarose gel). Finally, forward and reverse sequencing was performed by MACROGEN (Madrid, Spain), using standard Sanger sequencing method (Sanger & Coulson, 1975).

### 2.3 Genetic analysis

The resulting sequences were edited for quality trimming and primer removal using Geneious Primer 2022.2.2 (https://www.geneious.com). The cleaned sequences were aligned and manually checked for any possible error in base calling. Next, a consensus sequence was generated with the default parameters. Finally, sequences were identified with the best match identity (>97%) using nBlast implemented in Geneious Primer to search in GenBank databases.

The COI sequence of the marbled crayfish collected in Northern Spain was aligned with a set of 39 previously published sequences, deposited in GenBank, belonging to *P. alleni*, *P. clarkii*, *P. virginalis* and *P. fallax*, and a sequence of *F. limosus* which was used as an outgroup (GB Access Numbers in Figure 3). We verified the GenBank species name and associated notes and reviewed the original publications to confirm the identity of the species to which each sequence belonged. All sequences were analysed for phylogenetic analyses under maximum likelihood by using RaxML software (Stamatakis, 2014) implemented in Geneious Primer. The ModelTest software included in the PAUP pipeline was used to predict the nucleotide substitution model showing the best AIC and BIC scores. A maximum‐likelihood tree was generated using Rapid Bootstrapping Algorithm and a search was conducted for the best‐scoring tree using the general time reversible model (GTR + G + I) of molecular evolution with 10,000 bootstrap replicates. A consensus tree was generated with a 10% of burning and a 50% node support threshold value for bootstrapping but interpreted as significant nodes from a minimum value of 70%.

## 3 RESULTS

**FIGURE 2:**
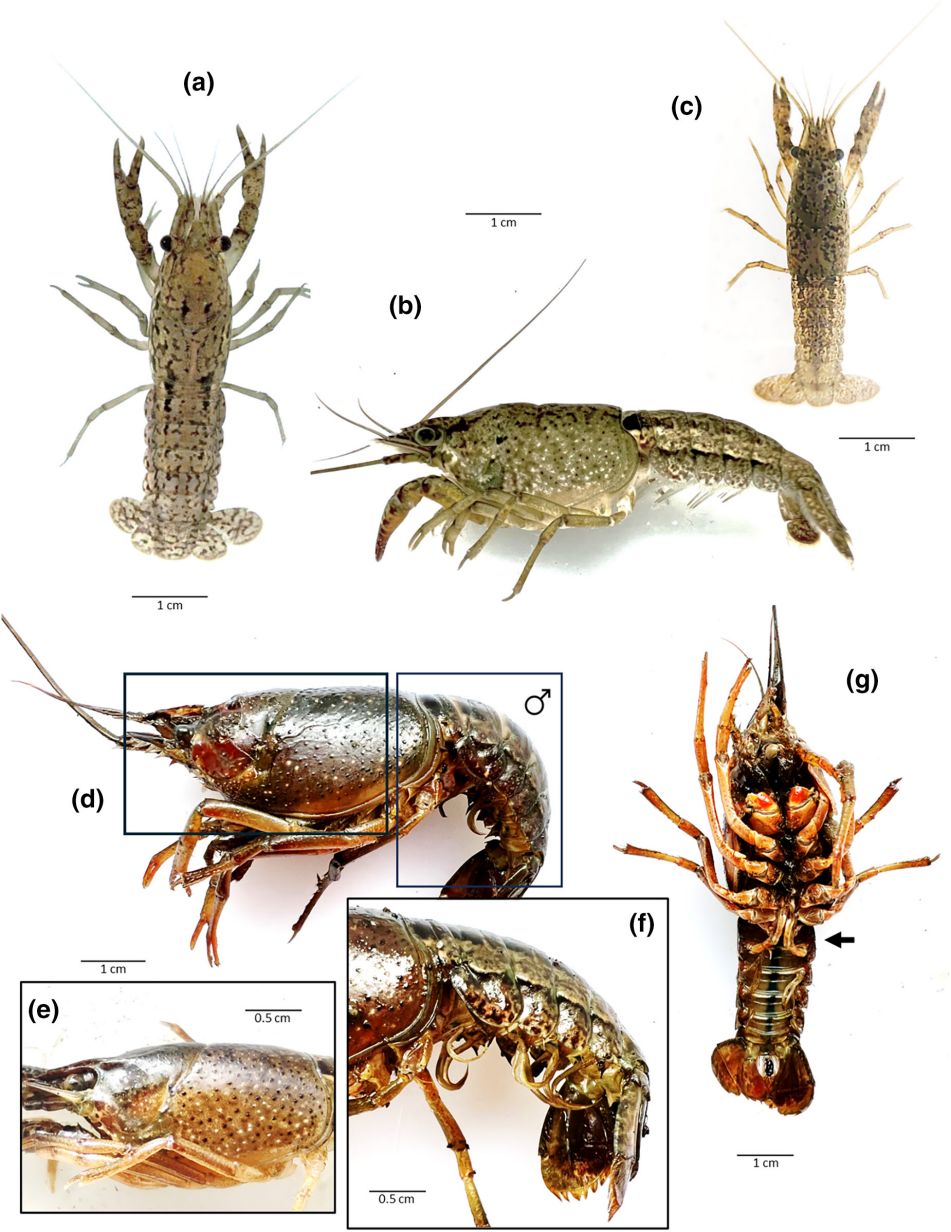
*Procambarus* live specimens from the Nalón River (northern Spain). *Procambarus virginalis* (a–c): dorsal view (a), lateral view of the same specimen (b) and dorsal view of a smaller specimen (c). *Procambarus clarkii* (d‐e): lateral view of male specimen (d), detailed view of the carapace (e), detailed view of the abdomen (f) and ventral view of the same specimen (g). Arrow points to the male gonopods.

Morphological examination of the collected specimens showed that they were consistent with the original diagnosis of *P. virginalis* by Lyko (2017). Specimens ranged in total length from 59 to 63 mm, with the carapace (27–29 mm) slightly shorter than the abdomen (32–34 mm). Live specimens have a marmorated colouration, appearing brownish or greenish, featuring dark‐spotted pattern on the lateral cephalothorax and abdomen (Figure 2a–c). Ethanol‐stored specimens turned whitish to cream coloured. No specimens with eggs were caught.

**FIGURE 3:**
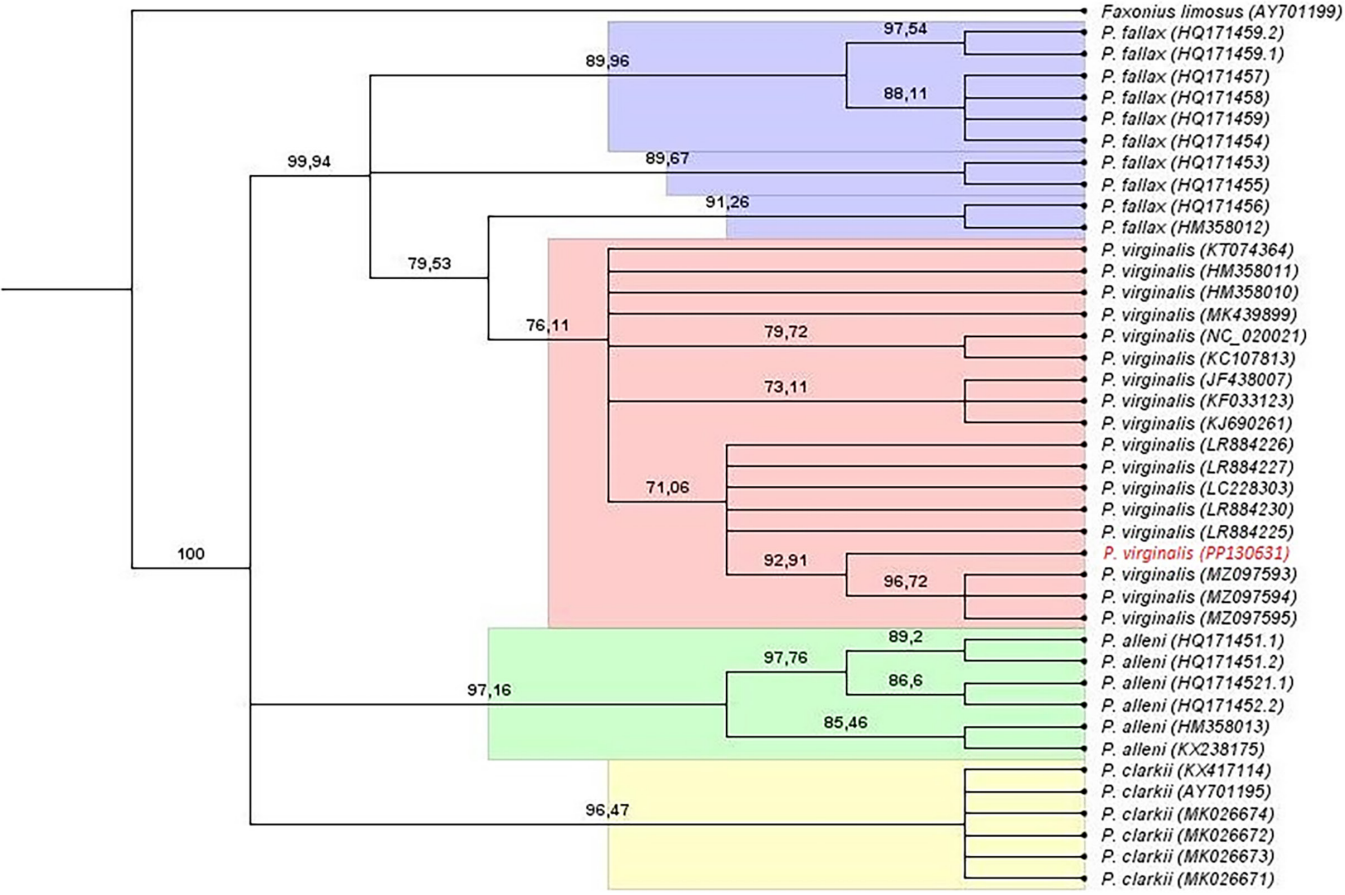
Cladogram of the genus *Procambarus*. Maximum‐likelihood method based on COI sequences using the GTR + G + I model with 10,000 bootstrap replicates and 10% of burning was considered to interpret significant nodes, setting a minimum value of 70%. The sequence from this work is shown in red.

A 619 bp fragment of COI gene was successfully sequenced from one studied specimen and deposited in GenBank under the Accession Number PP130631. The Blast identification engine identified the sequences as *P. virginalis* with 99.8%–100% pairwise identity with 76 specimens. The remaining hits were with other species of the genus *Procambarus* with a similarity of 96% or below. The phylogenetic analyses assessed using COI sequences from different species of *Procambarus* genus clearly show how the sequence of the Iberian specimen clusters with the sequences identified in GenBank as *P. virginalis*. The node clustering them all together has 76.11% bootstrapping support (Figure 3).

At the Nalón River, *P. virginalis* co‐occurred with *P. clarkii* of different colour morphs, outstanding one with marmorated colouration on cephalothorax and abdomen (Figure 2d–g). *Procambarus clarkii* was the most abundant crayfish species.

**FIGURE 4:**
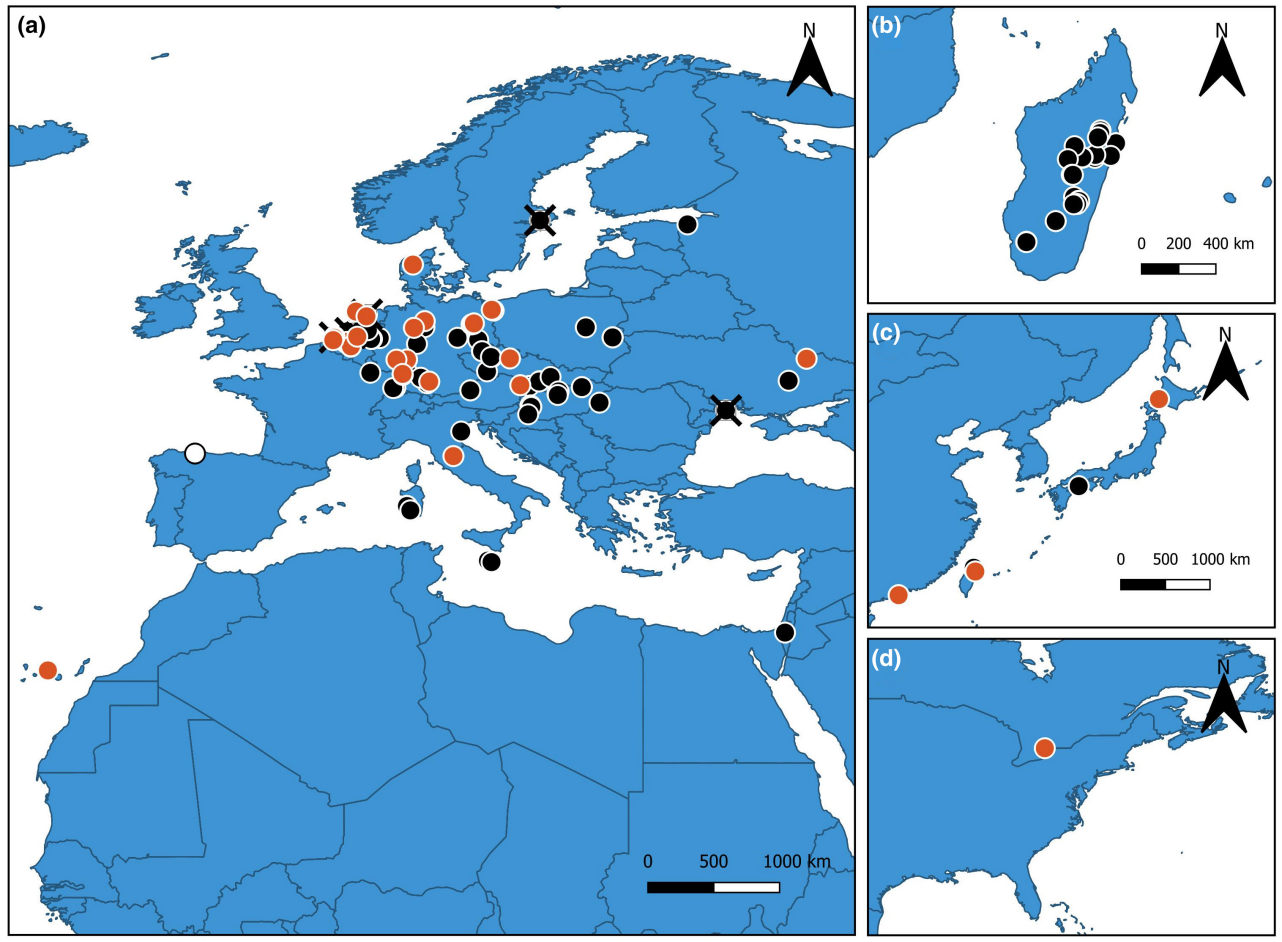
Geographical distribution of *Procambarus virginalis* in Europe (a), Africa (b), Asia (c) and North America (d). The black dots indicate established populations while the red dots show feral populations whose status is still unknown. The white dot indicates the new locality for the species and the black crosses indicate populations that have been successfully eradicated. Adapted from https://sites.google.com/view/marmorkrebs/.

As mentioned in the introductory part, after the first reports of *P. virginalis* in the wild around 2003 from northern Europe and Madagascar, it has been established in different countries and regions of Europe, Asia, Africa and North America. Notably, new species records have increased dramatically over the last decade. We used the available information to give a synopsis of the recent records of the marbled crayfish in the wild, including newly discovered specimens from northern Spain and the Canary Islands. An updated geographical distribution of *P. virginalis*, together with data about the status of the recorded populations (established/unknown/eradicated), is provided in Figure 4.

## 4 DISCUSSION

This study reports the first occurrence of marbled crayfish *P. virginalis* in the Iberian inland waters. Although born in an aquarium, the phylogeographic origin of marbled crayfish seems to be from the Everglades subpopulation of *P. fallax* (Gutekunst et al., 2021). To date, this species is widely distributed in temperate areas of central and southern Europe and Asia, as well as in certain subtropical areas. It has established wild populations in several countries over the world including Madagascar, Germany, Netherlands, Belgium, Austria, Poland, Slovakia, Czech Republic, Hungary, Romania, Italy, Israel, France, Malta, Croatia, Ukraine and Japan; with feral populations not yet established in Denmark, Estonia, Canada and China (Figure 4) (Chucholl et al., 2012; Jones et al., 2009; Kawai et al., 2009; Liptak et al., 2016; Lőkkös et al., 2016; Marzano et al., 2009; Novitsky & Son, 2016; Patoka et al., 2016). Recently, an introduced population has been reported in the Canary Islands by a national press release, although confirmation is still needed. Although the introduction pathway through which this species has appeared in northern Spain remains unknown, it is likely the result of an intentional release or escape of animals kept in captivity (Oficialdegui et al., 2023). Indeed, although the marbled crayfish sale has been restricted, it can be found in pet trade, which has been considered as its main introduction pathway in other countries (Faulkes, 2010; Lipták et al., 2023).

As pointed out by Jones et al. (2009), the marbled crayfish can be considered ‘the perfect invader’. *Procambarus virginalis* is the only of the nearly 700 known crayfish species (Crandall & De Grave, 2017), whose reproductive pathway is obligatory apomictic parthenogenesis (Scholtz et al., 2003; Vogt, 2008). Not only does this way of reproduction make its establishment in the wild more favourable, but it also presents other reproductive traits that give it adaptive advantages over other species. As an example, it has been observed that it presents higher fecundity and much shorter egg incubation periods (20–42 days) compared to the European native species, or even presents an early maturity, reaching around 5–7 months compared to other native and introduced species, whose maturity is around 1–5 years depending on the species (Jones et al., 2009). Furthermore, in sympatry with other invasive crayfish species, marbled crayfish success can be largely attributed to its relatively rapid growth and its tendency to reproduce early and frequently (Kouba et al., 2021). As mentioned above, this species has been found inhabiting the same spaces as *P. clarkii*. Juveniles of this species may have marbled patterns like *P. virginalis*, but, notably, five *P. clarkii* large specimens (of total length over 8 cm) with marmorated colouration were found in the same area (Figure 2d–g). Although the probability of hybridization between both species is very low, due to the triploid genome of *P. virginalis* that represents a major cytogenetic barrier for meiotic chromosome segregation (Lyko, 2017; Martin et al., 2016), this fact may lead to a species misidentification in the field if it is only based on a visual identification, without a detailed anatomical and genetic analysis.

Modelling work by Feria and Faulkes (2011) already predicted the high environmental suitability of the northern and eastern fringe of Spain for *P. virginalis*. In particular, the lower area of the Nalón River, where this species was found, has very favourable habitats for its implantation and settlement. The introduction of the marbled crayfish in an area previously occupied by huge established populations of other invasive crayfish species (e.g. *P. clarkii* or *Pa. leniusculus*) does not seem to alter the trophic relationships in these ecosystems, but its presence can increase other pressures on freshwater ecosystems (Chucholl & Chucholl, 2021; Linzmaier et al., 2020; Lipták et al., 2019; Veselý et al., 2021). On the other hand, it has been confirmed that *P. virginalis* (as the widespread *P. clarkii* and *Pa. leniusculus*) is a vector of the parasitic fungus *Aphanomyces astaci*, causative agent of the crayfish plague (Keller et al., 2014; Mrugała et al., 2015). This disease is considered responsible for the decline in numerous populations of endangered Eurasian crayfish species, including *A. fulcisianus* (Martín‐Torrijos et al., 2019).

The potential impact of the marbled crayfish could be considered like that of other invasive crayfish species already present in the Iberian freshwater ecosystems, such as *P. clarkii* and *Pa. leniusculus*. Nevertheless, these impacts could be increased by the ease of reproduction and dispersal of *P. virginalis*. Few data are known on the impact of this species on the native fauna and the ecosystems it manages to colonise. There are only experimental data on its predation on gastropod molluscs of the families Planorbidae (*Biomphalaria glabrata*) and Bulimidae (*Bulimus truncatus*) (Faiad et al., 2023). Although neither of these species is native to the habitat where *P. virginalis* is herein reported, there are other indigenous species of planorbids (e.g. *Gyraulus parvus*), Physidae (e.g. *Physella acuta*) and Lymnaeidae (e.g. *Radix* sp. and *Ampullaceana* sp.) that could potentially be preyed by this species. Furthermore, the Nalón‐Narcea Basin hosts one of the most important populations of the freshwater pearl mussel *Margaritifera margaritifera* (Álvarez‐Claudio et al., 2000; Lopes‐Lima et al., 2015; Perea et al., 2022), an endangered species included in the Spanish Catalogue of Threatened Species (Order AAA/1351/2016 modifying the Royal Decree 139/2011) and the European Habitats Directive (Council Directive 92/43/EEC). Likewise, the lower reaches of the Nalón River are also reproductive habitats for the threatened sea lamprey (*Petromyzon marinus*) (Mateus et al., 2012; Mota et al., 2016). As has been demonstrated, the presence of invasive crayfish can cause serious problems in *M. margaritifera* populations (Morales, 2023) and *P. marinus* (Hume et al., 2021; Smith & Marsden, 2009), so the presence of *P. virginalis* can cause similar effects of direct predation of adults, eggs and/or juveniles, triggering serious problems in the dynamics of aquatic communities.

Due to the impacts described above, the marbled crayfish was listed in the Invasive Alien Species of Union concern (i.e. Union list, EU Regulation No. 1143/2014). Additionally, it was classified as a high‐risk species according to the Freshwater Invertebrate Invasiveness Scoring Kit (Chucholl, 2016) and ranked among the top 10 invasive alien species listed in the Alert List during a recent horizon scan exercise for Iberian waters (Oficialdegui et al., 2023). However, on account of its later description, it was not included in the Spanish Catalogue of Invasive Alien Species (Royal Decree 630/2013). As a result of its inclusion in the Union List, this species is subject to restrictions, and its trade and breeding are prohibited. The currently available data cannot confirm the establishment of a population in northern Spain, as only a few individuals were found, and no gravid females were detected. Even so, the situation is worrisome as the presence of a single individual in the wild with optimal conditions may trigger the establishment of a population with serious ecological problems to biodiversity and ecosystems. We therefore strongly urge the authorities to implement rapid management measures and monitor the status of native threatened taxa (e.g. *M. margaritifera* and *P. marinus* populations), as well as the evolution of the marbled crayfish. Large demographic assemblage of *P. virginalis* could be reached relatively quickly and, reached that point, eradication or control actions may be extremely difficult to carry out efficiently. Finally, this study confirms the importance of early warning systems for exotic species, keeping the population, forest guards and field technicians informed about potential invasive species to execute a rapid and effective response.

## AUTHOR CONTRIBUTIONS

**Omar Sánchez:** Formal analysis (lead); investigation (equal); methodology (equal); writing – original draft (lead); writing – review and editing (equal). **Francisco J. Oficialdegui:** Conceptualization (equal); formal analysis (equal); investigation (equal); writing – original draft (equal); writing – review and editing (equal). **Antonio Torralba‐Burrial:** Conceptualization (lead); formal analysis (equal); investigation (equal); writing – original draft (equal); writing – review and editing (equal). **Ramón Arbesú:** Resources (equal); writing – review and editing (equal). **José María Valle‐Artaza:** Resources (equal); writing – review and editing (equal). **Ángel Fernández‐González:** Resources (equal); writing – review and editing (equal). **Alba Ardura:** Formal analysis (equal); methodology (equal); writing – review and editing (equal). **Andrés Arias:** Conceptualization (lead); formal analysis (equal); funding acquisition (lead); investigation (equal); supervision (lead); writing – original draft (lead); writing – review and editing (lead).

## CONFLICT OF INTEREST STATEMENT

The authors declare no conflict of interest.

## Data Availability

All the genetic data are available at GenBank using the accession numbers provided in Figure 3.

## References

[ece311362-bib-0001] Acevedo‐Limón, L. , Oficialdegui, F. J. , Sánchez, M. I. , & Clavero, M. (2020). Historical, human, and environmental drivers of genetic diversity in the red swamp crayfish (*Procambarus clarkii*) invading the Iberian Peninsula. Freshwater Biology, 65(8), 1460–1474. 10.1111/fwb.13513

[ece311362-bib-0002] Álvarez‐Claudio, C. , García‐Rovés, P. , Ocharan, R. , Cabal, J. A. , Ocharan, F. J. , & Alvarez, M. A. (2000). A new record of the freshwater pearl mussel *Margaritifera margaritifera* L. (Bivalvia, Unionoida) from the river Narcea (Asturias, north‐western Spain). Aquatic Conservation: Marine and Freshwater Ecosystems, 10(2), 93–102. 10.1002/(SICI)1099-0755(200003/04)10:2<93::AID-AQC392>3.0.CO;2-4

[ece311362-bib-0003] Arias, A. , & Torralba‐Burrial, A. (2021). First record of the redclaw crayfish *Cherax quadricarinatus* (Von Martens, 1868) on the Iberian Peninsula. Limnetica, 40, 33–42. 10.23818/limn.40.03

[ece311362-bib-0004] Benejam, L. , Saura‐Mas, S. , & Saperas, A. (2011). First record of the spiny‐cheek crayfish *Orconectes limosus* (Rafinesque, 1817) introduced to the Iberian Peninsula. Aquatic Invasions, 6, 111–113. 10.3391/ai.2011.6.S1.025

[ece311362-bib-0005] Carlton, J. T. (1996). Biological invasions and cryptogenic species. Ecology, 77(6), 1653–1655.

[ece311362-bib-0006] Chiatante, G. , Pellitteri‐Rosa, D. , Torretta, E. , Marzano, F. N. , & Meriggi, A. (2021). Indicators of biodiversity in an intensively cultivated and heavily human modified landscape. Ecological Indicators, 130, 108060. 10.1016/j.ecolind.2021.108060

[ece311362-bib-0007] Chucholl, C. (2016). Marbled crayfish gaining ground in Europe: The role of the pet trade as invasion pathway. In T. Kawai , Z. Faulkes , & G. Scholtz (Eds.), Freshwater crayfish: Global overview (pp. 83–114). CRC Press. 10.1201/b18723-8

[ece311362-bib-0008] Chucholl, C. , Morawetz, K. , & Groß, H. (2012). The clones are coming–strong increase in Marmorkrebs [*Procambarus fallax* (Hagen, 1870) f. *virginalis*] records from Europe. Aquatic Invasions, 7(4), 511–519. 10.3391/ai.2012.7.4.008

[ece311362-bib-0010] Chucholl, F. , & Chucholl, C. (2021). Differences in the functional responses of four invasive and one native crayfish species suggest invader‐specific ecological impacts. Freshwater Biology, 66(11), 2051–2063. 10.1111/fwb.13813

[ece311362-bib-0011] Clavero, M. , & Centeno‐Cuadros, A. (2016). Multiple, solid evidence support that *Austropotamobius italicus* is not native to Spain. Organisms Diversity & Evolution, 16, 715–717. 10.1007/s13127-016-0296-0

[ece311362-bib-0012] Clavero, M. , Nores, C. , Kubersky‐Piredda, S. , & Centeno‐Cuadros, A. (2016). Interdisciplinarity to reconstruct historical introductions: Solving the status of cryptogenic crayfish. Biological Reviews, 91(4), 1036–1049. 10.1111/brv.12205 26177420

[ece311362-bib-0013] Crandall, K. A. , & De Grave, S. (2017). An updated classification of the freshwater crayfishes (Decapoda: Astacidea) of the world, with a complete species list. Journal of Crustacean Biology, 37(5), 615–653. 10.1093/jcbiol/rux070

[ece311362-bib-0014] Emery‐Butcher, H. E. , Beatty, S. J. , & Robson, B. J. (2020). The impacts of invasive ecosystem engineers in freshwaters: A review. Freshwater Biology, 65(5), 999–1015. 10.1111/fwb.13479

[ece311362-bib-0015] Faiad, S. M. , Williams, M. A. , Goodman, M. , Sokolow, S. , Olden, J. D. , Mitchell, K. , Andriantsoa, R. , Gordon‐Jones, J. P. , Andriamaro, L. , Ravoniarimbinina, P. , Rasamy, J. , Ravelomanana, T. , Ravelotafita, S. , Ravo, R. , Rabinowitz, P. , De Leo, G. A. , & Wood, C. L. (2023). Temperature affects predation of schistosome‐competent snails by a novel invader, the marbled crayfish *Procambarus virginalis* . PLoS One, 18(9), e0290615. 10.1371/journal.pone.0290615 37703262 PMC10499222

[ece311362-bib-0016] Faulkes, Z. (2010). The spread of the parthenogenetic marbled crayfish, Marmorkrebs (*Procambarus* sp.), in the north American pet trade. Aquatic Invasions, 5(4), 447–450. 10.3391/ai.2010.5.4.16

[ece311362-bib-0017] Feria, T. P. , & Faulkes, Z. (2011). Forecasting the distribution of Marmorkrebs, a parthenogenetic crayfish with high invasive potential, in Madagascar, Europe, and North America. Aquatic Invasions, 6(1), 55–67. 10.3391/ai.2011.6.1.07

[ece311362-bib-0018] Filonzi, L. , Ardenghi, A. , Rontani, P. M. , Voccia, A. , Ferrari, C. , Papa, R. , Bellin, N. , & Nonnis Marzano, F. (2023). Molecular barcoding: A tool to guarantee correct seafood labelling and quality and preserve the conservation of endangered species. Food, 12(12), 2420. 10.3390/foods12122420 PMC10297432 37372635

[ece311362-bib-0019] Folmer, O. , Black, M. , Hoeh, W. , Lutz, R. , & Vrijenhoek, R. (1994). DNA primers for amplification of mitochondrial cytochrome c oxidase subunit I from diverse metazoan invertebrates. Molecular Marine Biology and Biotechnology, 3, 294–299. 7881515

[ece311362-bib-0021] Gutekunst, J. , Maiakovska, O. , Hanna, K. , Provataris, P. , Horn, H. , Wolf, S. , Skelton, C. E. , Dorn, N. J. , & Lyko, F. (2021). Phylogeographic reconstruction of the marbled crayfish origin. Communications Biology, 4(1), 1096. 10.1038/s42003-021-02609-w 34535758 PMC8448756

[ece311362-bib-0022] Hume, J. B. , Almeida, P. R. , Buckley, C. M. , Criger, L. A. , Madenjian, C. P. , Robinson, K. F. , Wang, C. J. , & Muir, A. M. (2021). Managing native and non‐native sea lamprey (*Petromyzon marinus*) through anthropogenic change: A prospective assessment of key threats and uncertainties. Journal of Great Lakes Research, 47, S704–S722. 10.1016/j.jglr.2020.08.015

[ece311362-bib-0024] Jones, J. P. , Rasamy, J. R. , Harvey, A. , Toon, A. , Oidtmann, B. , Randrianarison, M. H. , Raminosoa, N. , & Ravoahangimalala, O. R. (2009). The perfect invader: A parthenogenic crayfish poses a new threat to Madagascar's freshwater biodiversity. Biological Invasions, 11, 1475–1482. 10.1007/s10530-008-9334-y

[ece311362-bib-0025] Kawai, T. , Scholtz, G. , Morioka, S. , Ramanamandimby, F. , Lukhaup, C. , & Hanamura, Y. (2009). Parthenogenetic alien crayfish (Decapoda: Cambaridae) spreading in Madagascar. Journal of Crustacean Biology, 29(4), 562–567. 10.1651/08-3125.1

[ece311362-bib-0026] Keller, N. S. , Pfeiffer, M. , Roessink, I. , Schulz, R. , & Schrimpf, A. (2014). First evidence of crayfish plague agent in populations of the marbled crayfish (*Procambarus fallax* forma *virginalis*). Knowledge and Management of Aquatic Ecosystems, 414, 15. 10.1051/kmae/2014032

[ece311362-bib-0027] Kouba, A. , Lipták, B. , Kubec, J. , Bláha, M. , Veselý, L. , Haubrock, P. J. , Oficialdegui, F. J. , Niksirat, H. , Patoka, J. , & Buřič, M. (2021). Survival, growth, and reproduction: Comparison of marbled crayfish with four prominent crayfish invaders. Biology, 10(5), 422. 10.3390/biology10050422 34068504 PMC8151088

[ece311362-bib-0028] Kouba, A. , Oficialdegui, F. J. , Cuthbert, R. N. , Kourantidou, M. , South, J. , Tricarico, E. , Gozlan, R. E. , Courchamp, F. , & Haubrock, P. J. (2022). Identifying economic costs and knowledge gaps of invasive aquatic crustaceans. Science of the Total Environment, 813, 152325. 10.1016/j.scitotenv.2021.152325 34971690

[ece311362-bib-0029] Linzmaier, S. M. , Musseau, C. , Matern, S. , & Jeschke, J. M. (2020). Trophic ecology of invasive marbled and spiny‐cheek crayfish populations. Biological Invasions, 22(11), 3339–3356. 10.1007/s10530-020-02328-z

[ece311362-bib-0030] Liptak, B. , Mrugała, A. , Pekarik, L. , Mutkovič, A. , Gruľa, D. , Petrusek, A. , & Kouba, A. (2016). Expansion of the marbled crayfish in Slovakia: Beginning of an invasion in the Danube catchment? Journal of Limnology, 75(2), 305–312. 10.4081/jlimnol.2016.1313

[ece311362-bib-0031] Lipták, B. , Veselý, L. , Ercoli, F. , Bláha, M. , Buřič, M. , Ruokonen, T. , & Kouba, A. (2019). Trophic role of marbled crayfish in a lentic freshwater ecosystem. Aquatic Invasions, 14(2), 299–309. 10.3391/ai.2019.14.2.0

[ece311362-bib-0032] Lipták, B. , Zorić, K. , Patoka, J. , Kouba, A. , & Paunović, M. (2023). The aquarium pet trade as a source of potentially invasive crayfish species in Serbia. Biologia, 78, 2147–2155. 10.1007/s11756-023-01347-0

[ece311362-bib-0033] Lodge, D. M. , Deines, A. , Gherardi, F. , Yeo, D. C. , Arcella, T. , Baldridge, A. K. , Barnes, M. A. , Chadderton, W. L. , Feder, J. L. , Gantz, C. A. , Howard, G. W. , Jerde, C. L. , Peters, B. W. , Peters, J. A. , Sargent, L. W. , Turner, C. R. , Wittmann, M. E. , & Zeng, Y. (2012). Global introductions of crayfishes: Evaluating the impact of species invasions on ecosystem services. Annual Review of Ecology, Evolution, and Systematics, 43, 449–472. 10.1146/annurev-ecolsys-111511-103919

[ece311362-bib-0034] Lőkkös, A. , Müller, T. , Kovács, K. , Várkonyi, L. , Specziár, A. , & Martin, P. (2016). The alien, parthenogenetic marbled crayfish (Decapoda: Cambaridae) is entering Kis‐Balaton (Hungary), one of Europe's most important wetland biotopes. Knowledge and Management of Aquatic Ecosystems, 417, 16. 10.1051/kmae/2016003

[ece311362-bib-0035] Lopes‐Lima, M. , Sousa, R. , Geist, J. , Aldridge, D. , Araujo, R. , Bergengren, J. , Bespalaya, Y. , Bódis, E. , Burlakova, L. , Van Damme, D. , Douda, K. , Froufe, E. , Georgiev, D. , Gumpinger, C. , Karatayev, A. , Kebapçi, Ü. , Killeen, I. , Lajtner, J. , Larsen, B. M. , … Zogaris, S. (2015). Conservation status of freshwater mussels in Europe: State of the art and future challenges. Biological Reviews, 92(1), 572–607. 10.1111/brv.12244 26727244

[ece311362-bib-0036] Lyko, F. (2017). The marbled crayfish (Decapoda: Cambaridae) represents an independent new species. Zootaxa, 4363(4), 544–552. 10.11646/zootaxa.4363.4.6 29245391

[ece311362-bib-0037] Martin, P. , Dorn, N. J. , Kawai, T. , van der Heiden, C. , & Scholtz, G. (2010). The enigmatic Marmorkrebs (marbled crayfish) is the parthenogenetic form of *Procambarus fallax* (Hagen, 1870). Contributions to Zoology, 79(3), 107–118. 10.1163/18759866-07903003

[ece311362-bib-0038] Martin, P. , Thonagel, S. , & Scholtz, G. (2016). The parthenogenetic Marmorkrebs (malacostraca: Decapoda: Cambaridae) is a triploid organism. Journal of Zoological Systematics and Evolutionary Research, 54(1), 13–21. 10.1111/jzs.12114

[ece311362-bib-0039] Martínez‐Ríos, M. , Martín‐Torrijos, L. , Casabella‐Herrero, G. , Tedesco, P. , Machordom, A. , & Diéguez‐Uribeondo, J. (2023). On the conservation of white‐clawed crayfish in the Iberian Peninsula: Unraveling its genetic diversity and structure, and origin. PLoS One, 18(10), e0292679. 10.1371/journal.pone.0292679 37831691 PMC10575519

[ece311362-bib-0040] Martín‐Torrijos, L. , Kokko, H. , Makkonen, J. , Jussila, J. , & Diéguez‐Uribeondo, J. (2019). Mapping 15 years of crayfish plague in the Iberian Peninsula: The impact of two invasive species on the endangered native crayfish. PLoS One, 14(8), e0219223. 10.1371/journal.pone.0219223 31393870 PMC6687115

[ece311362-bib-0041] Marzano, F. N. , Scalici, M. , Chiesa, S. , Gherardi, F. , Piccinini, A. , & Gibertini, G. (2009). The first record of the marbled crayfish adds further threats to fresh waters in Italy. Aquatic Invasions, 4(2), 401–404. 10.3391/ai.2009.4.2.19

[ece311362-bib-0042] Matallanas, B. , Ochando, M. D. , Alonso, F. , & Callejas, C. (2016). Update of genetic information for the white‐clawed crayfish in Spain, with new insights into its population genetics and origin. Organisms Diversity & Evolution, 16, 533–547. 10.1007/s13127-016-0268-4

[ece311362-bib-0043] Mateus, C. S. , Rodríguez‐Muñoz, R. , Quintella, B. R. , Alves, M. J. , & Almeida, P. R. (2012). Lampreys of the Iberian Peninsula: Distribution, population status and conservation. Endangered Species Research, 16(2), 183–198. 10.3354/esr00405

[ece311362-bib-0044] Morales, J. (2023). The signal crayfish *Pacifastacus leniusculus* (Dana, 1852) (Crustacea, Decapoda) is threatening the near future of *Margaritifera margaritifera* Linnaeus, 1758 (Bivalvia, Unionoida) in the Negro River (NW Zamora, Spain). Animal Biodiversity and Conservation, 46(2), 165–171. 10.32800/abc.2023.46.0165

[ece311362-bib-0045] Mota, M. , Rochard, E. , & Antunes, C. (2016). Status of the diadromous fish of the Iberian Peninsula: Past, present and trends. Limnetica, 35(1), 1–18. 10.23818/limn.35.01

[ece311362-bib-0046] Mrugała, A. , Kozubíková‐Balcarová, E. , Chucholl, C. , Cabanillas‐Resino, S. , Viljamaa‐Dirks, S. , Vukić, J. , & Petrusek, A. (2015). Trade of ornamental crayfish in Europe as a possible introduction pathway for important crustacean diseases: Crayfish plague and white spot syndrome. Biological Invasions, 17, 1313–1326. 10.1007/s10530-014-0795-x

[ece311362-bib-0047] Novitsky, R. A. , & Son, M. O. (2016). The first records of Marmorkrebs [*Procambarus fallax* (Hagen, 1870) f. *virginalis*] (Crustacea, Decapoda, Cambaridae) in Ukraine. Ecologica Montenegrina, 5, 44–46. 10.37828/em.2016.5.8

[ece311362-bib-0048] Oficialdegui, F. J. , Zamora‐Marín, J. M. , Guareschi, S. , Anastácio, P. M. , García‐Murillo, P. , Ribeiro, F. , Miranda, R. , Cobo, F. , Gallardo, B. , García‐Berthou, E. , Boix, D. , Arias, A. , Cuesta, J. A. , Medina, L. , Almeida, D. , Banha, F. , Barca, S. , Biurrun, I. , Cabezas, M. P. , … Oliva‐Paterna, F. J. (2023). A horizon scan exercise for aquatic invasive alien species in Iberian inland waters. Science of the Total Environment, 869, 161798. 10.1016/j.scitotenv.2023.161798 36702272

[ece311362-bib-0049] Patoka, J. , Buřič, M. , Kolář, V. , Bláha, M. , Petrtýl, M. , Franta, P. , Tropek, R. , Kalous, L. , Petrusek, A. , & Kouba, A. (2016). Predictions of marbled crayfish establishment in conurbations fulfilled: Evidences from The Czech Republic. Biologia, 71, 1380–1385. 10.1515/biolog-2016-0164

[ece311362-bib-0050] Perea, S. , Mendes, S. L. , Sousa‐Santos, C. , Ondina, P. , Amaro, R. , Castro, J. , San‐Miguel, E. , Lima, C. S. , García, M. , Velasquez, V. , García‐Roves, P. , Fernández, D. , Araujo, R. , Sousa, V. C. , & Reis, J. (2022). Applying genomic approaches to delineate conservation strategies using the freshwater mussel *Margaritifera margaritifera* in the Iberian Peninsula as a model. Scientific Reports, 12(1), 16894. 10.1038/s41598-022-20947-5 36207367 PMC9546909

[ece311362-bib-0051] Reynolds, J. , Souty‐Grosset, C. , & Richardson, A. (2013). Ecological roles of crayfish in freshwater and terrestrial habitats. Freshwater Crayfish, 19(2), 197–218. 10.5869/fc.2013.v19-2.197

[ece311362-bib-0052] Sanger, F. , & Coulson, A. R. (1975). A rapid method for determining sequences in DNA by primed synthesis with DNA polymerase. Journal of Molecular Biology, 94(3), 441–448. 10.1016/0022-2836(75)90213-2 1100841

[ece311362-bib-0053] Scholtz, G. , Braband, A. , Tolley, L. , Reimann, A. , Mittmann, B. , Lukhaup, C. , Steuerwald, F. , & Vogt, G. (2003). Parthenogenesis in an outsider crayfish. Nature, 421(6925), 806. 10.1038/421806a 12594502

[ece311362-bib-0054] Smith, S. J. , & Marsden, J. E. (2009). Factors affecting sea lamprey egg survival. North American Journal of Fisheries Management, 29(4), 859–868. 10.1577/M07-196.1

[ece311362-bib-0055] Stamatakis, A. (2014). RAxML version 8: A tool for phylogenetic analysis and post‐analysis of large phylogenies. Bioinformatics, 30(9), 1312–1313. 10.1093/bioinformatics/btu033 24451623 PMC3998144

[ece311362-bib-0056] Vedia, I. , & Miranda‐Ferreiro, R. (2013). Review of the state of knowledge of crayfish species in the Iberian Peninsula. Limnetica, 32, 269–286. 10.23818/limn.32.22

[ece311362-bib-0057] Veselý, L. , Ruokonen, T. J. , Weiperth, A. , Kubec, J. , Szajbert, B. , Guo, W. , Ercoli, F. , Bláha, M. , Buřič, M. , Hämäläinen, H. , & Kouba, A. (2021). Trophic niches of three sympatric invasive crayfish of EU concern. Hydrobiologia, 848, 727–737. 10.1007/s10750-020-04479-5

[ece311362-bib-0058] Vogt, G. (2008). The marbled crayfish: A new model organism for research on development, epigenetics and evolutionary biology. Journal of Zoology, 276(1), 1–13. 10.1111/j.1469-7998.2008.00473.x

